# A protocol for a single center, randomized, controlled trial comparing the clinical efficacy of 3% diquafosol and 0.1% hyaluronic acid in diabetic patients with dry eye disease

**DOI:** 10.1186/s13063-023-07818-8

**Published:** 2023-12-12

**Authors:** Jiayan Chen, Yimeng Chen, Guanghao Qin, Liangzhe Li, Mingze Li, Yuan Cheng, Shuting Zhuang, Zhihui Li, Qing Zhang, Yi Wu, Lanting Yang, Salissou Moutari, Jonathan E. Moore, Ling Xu, Wei He, Sile Yu, Emmanuel Eric Pazo, Xingru He

**Affiliations:** 1 He Eye Specialist Hospital, Shenyang, China; 2 Jinzhou Medical University, Jinzhou, China; 3 Dalian Medical University, Shenyang, China; 4 Tianjin Medical University, Tianjin, China; 5 China Medical University, Shenyang, China; 6 Wenzhou Medical University, Wenzhou, China; 7 Mathematical Sciences Research Centre, School of Mathematics and Physics, Queen’s University Belfast, Belfast, UK; 8 Cathedral Eye Clinic, Belfast, UK; 9 He University, Shenyang, China

**Keywords:** Diabetic dry eye, Dry eye disease, Diquafosol, Hyaluronic acid, Randomized controlled trial

## Abstract

**Background:**

The global prevalence of diabetes mellitus (DM) continues to rise and 70% of diabetic individuals have dry eye disease (DED) that leads to subsequent abnormalities of the corneal epithelium, corneal nerves, tear film, or corneal endothelium. In addition, persons with diabetes produce fewer tear secretions than healthy individuals. While several anti-inflammatory drug-based therapies for dry eye in diabetic individuals are currently being administered, their efficacy has not been studied in detail. Therefore, the aim of this study was to compare the effectiveness of 3% diquafosol (DQS) vs 0.1% hyaluronic acid (HA) eye drops in diabetic dry eye patients.

**Methods:**

This triple-blind randomized, control trial will include 202 diabetic-related DED and will be assigned to DQS (*n* = 101) and HA (*n* = 101) one drop, six times per day for 8 weeks. Tear film lipid layer, non-invasive breakup time, conjunctivocorneal staining score, corneal sensitivity, tear MMP-9 levels, meibomian gland expression and quality, tear meniscus height, corneal nerves, immune/inflammatory cell change, conjunctival hyperemia, and ocular surface disease index questionnaire score will be assessed and compared at baseline, week 4, and week 8.

**Discussion:**

This study will be a standardized, scientific, clinical trial designed to evaluate the therapeutic effects and safety of DQS and HA for diabetic dry eye treatment.

**Trial registration:**

ClinicalTrials.govNCT05682547. Registered on December 05, 2022.

**Supplementary Information:**

The online version contains supplementary material available at 10.1186/s13063-023-07818-8.

## Administrative information

Please take note that the numerical values enclosed in braces within this protocol pertain to the corresponding item numbers in the SPIRIT checklist. The arrangement of the elements has been altered to cluster comparable items, as per the guidelines outlined in the SPIRIT 2013 Statement that defines standard protocol items for clinical trials. Please refer to (http://www.equator-network.org/reporting-guidelines/spirit-2013-statement-defining-standard-protocol-items-for-clinical-trials/) for further information.

**Table:** 

Title {1}	A protocol for a single center, randomized, controlled trial comparing the clinical efficacy of 3% diquafosol and 0.1 % hyaluronic acid in diabetic patients with dry eye disease
Trial registration {2a and 2b}	Clinicaltrials.NCT05682547
Protocol version {3}	Version 3 of Dec. 2022
Funding {4}	This study was entirely funded and sponsored by He Eye Specialist Hospital, Shenyang, China which includes, study design, data collection, analysis, interpretation, and manuscript writing. Non-financial support was received for the publication of this article.
Author details {5a}	**Jiayan Chen**. He Eye Specialist Hospital, Shenyang, China. **Yimeng Chen**. Jinzhou Medical University, Jinzhou China. **Quanghao Qin**. He Eye Specialist Hospital, Shenyang, China. **Liangzhe Li**. He Eye Specialist Hospital, Shenyang, China. **Mingze Li**. Dalian Medical University, Shenyang, China. **Yuan Cheng**. Dalian Medical University, Shenyang, China. **Shuting Zhuang**. Dalian Medical University, Shenyang, China. **Zhihui Li**. Dalian Medical University, Shenyang, China. **Qing Zhang**. Tianjin Medical University, Tianjin, China. **Yi Wu**. China Medical University, Shenyang, China. **Lanting Yang**. Wenzhou Medical University, Wenzhou, China. **Salissou Moutari**. Mathematical Sciences Research Centre, School of Mathematics and Physics, Queen's University Belfast, Belfast, UK. **Jonathan E Moore**. Cathedral Eye Clinic, Belfast, United Kingdom. **Ling Xu**. He Eye Specialist Hospital, Shenyang, China. **Wei He**. He Eye Specialist Hospital, Shenyang, China. **Sile Yu**. School of Public Health, He University, Shenyang, China. **Emmanuel Eric Pazo**. Department of Ophthalmology, He Eye Specialist Hospital, Shenyang, China. **Xingru He**. School of Public Health, He University, Shenyang, China.
Name and contact information for the trial sponsor {5b}	This is an investigator-initiated research, so the principal investigator acts as the sponsor. Emmanuel Eric Pazo (Principal Investigator). ericpazo@outlook.com
Role of sponsor {5c}	Investigator-initiated the research

## Introduction

### Background and rationale {6a}

Diabetes mellitus (DM) is a prevalent chronic metabolic illness that causes relative insulin insufficiency in target organs owing to pancreatic β-cell dysfunction and insulin resistance [1]. Shift to a sedentary lifestyle, an aging population and obesity have significantly contributed to the global rise in the prevalence of DM. In 2019, the prevalence of diabetes was documented to be 9.3% (463 million people) and in 2030 it is estimated to rise to 10.2% (578 million) and DM accounts for approximately 90% of all diabetic occurrences [2, 3]. Negative alterations to the tear film, corneal epithelium, corneal endothelium, and corneal nerves have been observed in 47–64% of patients with diabetes [4]. Ocular surface manifestation of signs and symptoms secondary to DM has been termed diabetic keratopathy (DK) [5]. DK has been documented to increase central corneal thickness [6], decrease endothelial cell density, lead to superficial punctate keratitis [7], delay and impede wound repair, and decrease corneal sensitivity due to neuropathy. Furthermore, DM patients have been noted to have compromised tear quantity and quality due to conjunctival goblet cell loss as documented by cytologic analysis. Goblet cells secrete mucin, which stabilizes the tear-film, minimizes tear evaporation, and reduces mechanical friction. Goblet cell loss in animal models suggests that it disrupts the ocular surface’s immune tolerance and increased the expression of inflammatory cytokines in the conjunctiva.

Diquafosol (DQS) is a dinucleotide polyphosphate which is a purinoceptor agonist; when administered to the ocular surface, it binds to P2Y2 receptors and stimulates mucin and tear secretion. The corneal epithelium, conjunctival epithelium, lacrimal gland ductal epithelium, meibomian gland sebaceous cells, and meibomian gland ductal cells all express the P2Y2 receptor. Subsequently, enhanced secretion of mucin and tear secretion due to DQS ophthalmic solution leads to stabilization of the tear film, minimizes tear evaporation, and reduces mechanical friction thereby protecting the corneal epithelium. Various reports have concluded that 3% DQS is effective in the treatment of dry eye disease and the year 2020's findings suggest that DQS improves corneal epithelial damage in DM rat model [8–10].

Around 0.1% hyaluronic acid (HA) used in artificial tears has been reported to promote corneal re-epithelium and improve corneal healing [11]. Additionally, HA has been reported to decrease the rate of tear evaporation and enhance the stability of tear film [12]. HA ophthalmic drops have emerged as a first-line treatment of choice for dry eye disease (DED) [13].

Although the significance of DED related to DM has been acknowledged and however, no clear guidelines for treatment options have been established or recommended [14]. Both DQS and HA eye drops are often prescribed to DED sufferers and are found to be clinically effective. However, these studies did not focus on evaluating the effectiveness of different eye-drop treatments in individuals with DED related to DM.

Therefore, the purpose is to assess and compare the effectiveness of DQS and HA topical eye drops on DM-related DED.

### Objectives {7}

The primary objective of this study is to perform a comparative efficacy assessment between DQS eye drop and HA eye drop in alleviating signs and symptoms of DED in diabetic patients.

### Trial design {8}

**Fig. 1:**
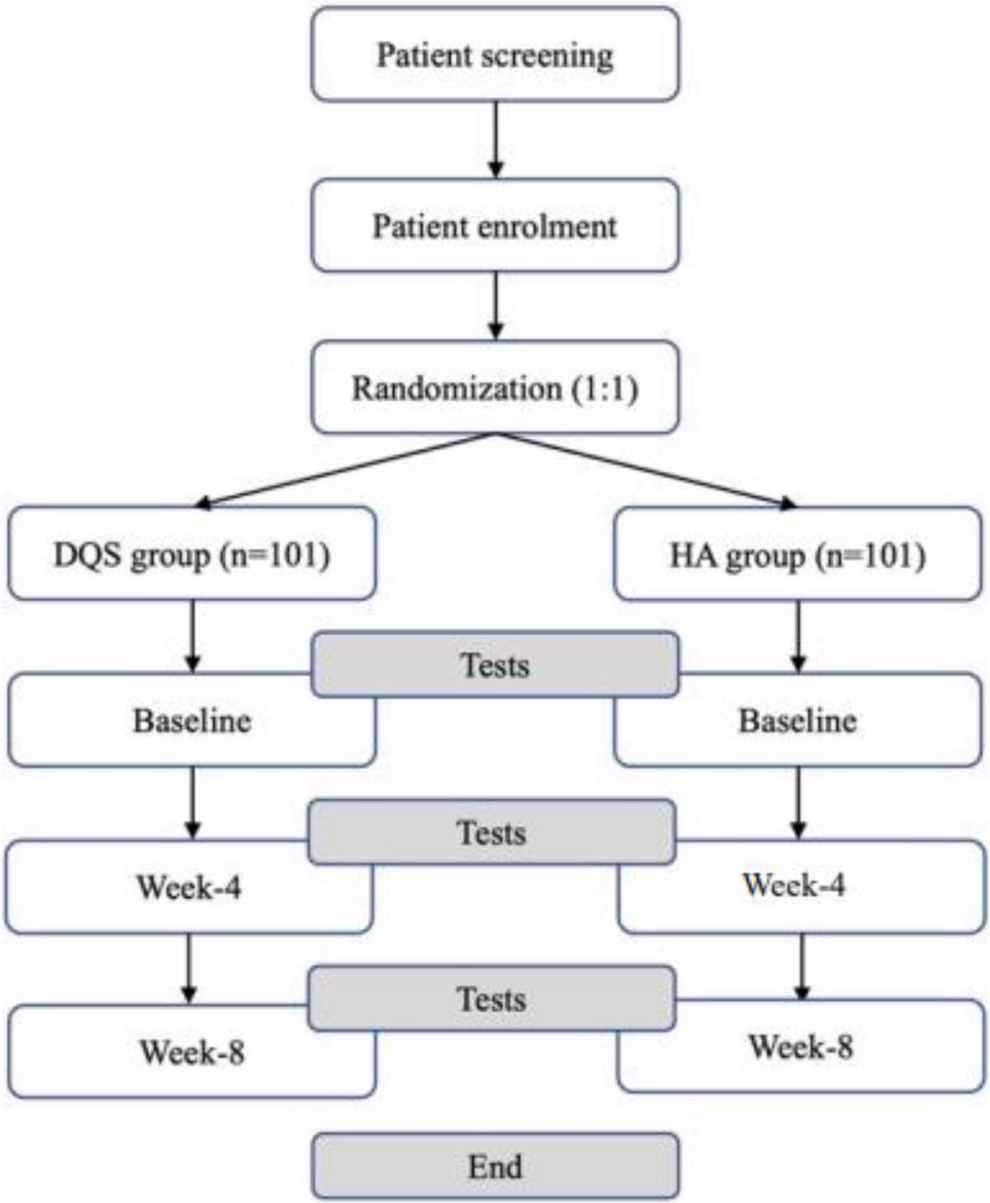
Study flowchart

This is a prospective, randomized, triple-blind, exploratory, controlled trial design study. Participants will be randomized (1:1) to the DQS group (*n* = 101) or HA group (*n* = 101). Participants in the DQS group will be administered one drop six times per day for 8 weeks (56 days), while the participants in the HA group will receive the assigned treatment over an 8-week period, one drop six times per day as depicted in Fig. 1.

## Methods: participants, interventions, and outcomes

### Study setting {9}

Diabetic patients diagnosed with DED will be recruited at the Department of Ophthalmology, He Eye Specialist Hospital (HESH), Shenyang, China.

### Eligibility criteria {10}

Inclusion criteria are as follows:

Age ≥18 years
(i) A1c≥6.5% (48 mmol/mol); (ii) fasting plasma glucose ≥126 mg/dL (7.0 mmol/L); and (iii) oral glucose tolerance test and random plasma glucose ≥200 mg/dL (11.1 mmol/L)
Able and willing to comply with the treatment/follow-up schedule.
Bilateral signs and symptoms of DED: (i) Ocular Surface Disease Index (OSDI) questionnaire ≥13, (ii) non-invasive tear breakup time (NITBUT) ≤ 5 seconds, and (iii) conjunctivocorneal staining score (CS) ≥3 points. The presence of two or more criteria was used to establish a positive DE diagnosis, based on the 2017 Asia Dry Eye Society criteria [15].

Exclusion criteria are as follows:

1. Patients with a history of hypersensitivity to any of the ingredients in DQS and artificial tear drops (ATDs).
2. Participants with systemic immune-mediated illnesses, such as secondary Sjögren’s syndrome or graft-versus-host disease.
3. Patients use topical medication(s) for the treatment of ocular disorders such as glaucoma or allergic conjunctivitis.
4. Previous ocular surgery or trauma.
5. 1-month history of blepharal and periorbital skin disease or allergies.
6. Severe dry eye with corneal epithelial defect.
7. Limbic keratitis.
8. Pterygium.
9. Corneal neovascularization.
10. Breastfeeding or pregnant women.
11. Rheumatic immune systemic diseases.
12. Herpes zoster infection.
13. Allergic to fluorescein.
14. Contact lens wearers.
15. Patients with active eye infections.
16. Patients with recurrent corneal erosions.
17. Patients with hereditary corneal disease.
18. Patients with punctal plugs or a history of surgical punctal occlusions.

### Informed consent {26a}

Trained and experienced clinicians will seek informed permission from prospective participants. After taking a detailed dry eye examination, if the potential participant meets the criteria and is willing to proceed to enrolment in the trial, they will sign and date informed consent forms. Here the potential participant will be fully briefed on the objective of the study and the contents of the informed consent form

### Additional consent provisions for collection and use of participant data and biological specimens {26b}

This trial does not involve collecting biological specimens.

### Interventions

#### Explanation for the choice of comparators {6b}

##### Study arm

Participants will use DQS eyedrops, a P2Y2 receptor agonist that promotes tear fluid and mucin secretion.

##### Control arm (active control)

Participants will utilize HA eyedrops as it has been shown to stimulate tear and mucus production on the ocular surface as a first-line treatment of choice for DED.

#### Intervention description {11a}

In this study, patients will receive either DQS (Diquas; Santen Pharmaceutical Co., Ltd., Osaka, Japan) or 0.1% hyaluronic acid (HA) artificial tears (preservative-free) eye drops for 8 weeks based on their randomized group allocation. The dosage for both drugs is 1 drop, 6 times per day. Two follow-up visits were performed on day 28 (4 weeks) and day 56 (8 weeks) in week 8 in both groups, comprehensive eye exams will be conducted by an ophthalmologist including primary outcomes, secondary outcomes, and safety evaluation.

#### Criteria for discontinuing or modifying allocated interventions {11b}

After enrollment in the study, participants will receive one drop of DQS or HA six times per day for 8 weeks (56 days). For very rare cases such as eye secretion, eye irritation, eye itching, congestion, and eye pain, participants will be advised to use the designated eye drops. Adverse events (AE) will be continuously monitored. In the case of an AE, participants will be informed about the severity of the event and the principal investigator (PI) will decide if participants can continue further. If participants consent and agree, they will be reminded daily regarding the administration of eye drops. Any queries regarding the study will be answered by trained clinical staff at HESH, Shenyang, China.

#### Strategies to improve adherence to interventions {11c}

Participants will be reminded by phone or email 1 week prior to their clinical visit; then, appointments will be scheduled in advance according to their availability. In order to improve adherence, patients will be given a medication (eyedrop) record booklet and their medication (eyedrop) status will be checked at each follow-up visit, and investigators will send a text message to remind the participants every day. In the event of non-compliance, such as absence, participants will be contacted by phone or email to confirm their willingness to continue or terminate the study.

#### Compliance

The research will be done on the basis of the intention to treat. On the basis of the eye-drop usage calendar, compliance with eye-drop use will be measured. There is no minimal compliance requirement for eye drop administration that would result in exclusion from the study; nevertheless, compliance will be monitored in statistical analyses and utilized as a gauge of the treatment's acceptability in accordance with the secondary goals. In addition to discussing evidence of overuse with participants, they will be instructed again on correct usage and compliance.

#### Relevant concomitant care permitted or prohibited during the trial {11d}

Any other systemic or topical medication, treatment, or therapy related to dry eye disease will be prohibited during the course of this study.

#### Provisions for post-trial care {30}

There is no anticipated harm and compensation for trial participation but participants who display signs and symptoms of deterioration in their dry eye status will be evaluated by the PI and be directed to their local dry eye center for further evaluation and necessary treatment.

#### Outcomes {12}

**Fig. 2:**
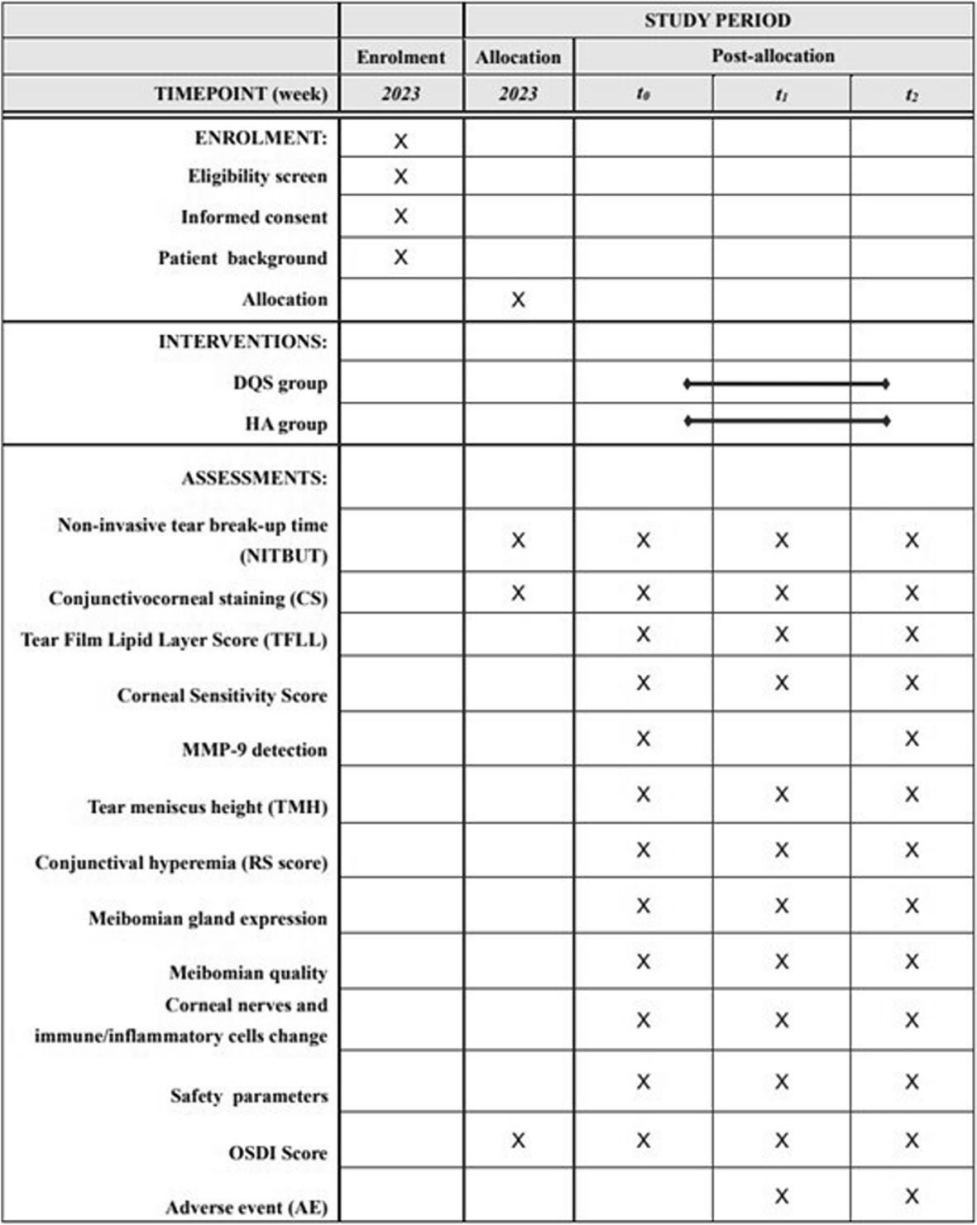
SPIRIT figure of enrolment, interventions, and assessments. *t*_*0*_, week 0; *t*_*1*_, week 4; *t*_*2*_, week 8

Figure 2 displays the timeline for data collection and site visits. The assessments will be conducted in accordance with a predetermined order. After obtaining the subjective symptom score using the  -OSDI) questionnaire [16], a physician will perform an in-person medical examination and lifestyle-related interview. Comprehensive eye exams will be conducted by an ophthalmologist. We plan to use primary and all the secondary outcomes (CS, TFLL score, TMH, RS score, MMP-9 detection, corneal sensitivity score, meibomian gland expression and quality, and corneal nerves and immune/inflammatory cells) to measure improvement which will be compared before (baseline) and after (day 28, day 56) between the groups, except for MMP-9 detection which is only at day 56.

#### Primary outcomes

NITBUT: The Keratograph 5M (Oculus, Germany) topographer will assess non-invasive tear breakup time. Three sequential readings will be captured, and the median value will be included in the final analysis. The median value will be recorded [17].

#### Secondary outcomes

CS: Fluorescein and lissamine staining of the ocular surface will be divided into three zones comprising nasal conjunctival, corneal, and temporal conjunctival areas. The staining score ranged from 0 to 3 for each zone, yielding a total score of 0–9 for the ocular surface [15].

Tear film lipid layer (TFLL) score: Tear film lipid layer interferometry will be assessed using DR-1 (Kowa, Nagoya, Japan). The results will be graded as follows: grade 1, somewhat gray color, uniform distribution; grade 2, somewhat gray color, nonuniform distribution; grade 3, a few colors, nonuniform distribution; grade 4, many colors, nonuniform distribution; grade 5, corneal surface partially exposed [18, 19].

Corneal Sensitivity Score: Corneal sensitivity score measured with a Cochet-Bonnet esthesiometer (in mm filament length); the measurements will be done 3 times at every assessment session and the median value will be recorded [20].

Matrix metalloproteinase 9 (MMP-9) detection: Inflammation Dry (Rapid Pathogen Screening Inc., Sarasota, FL, USA) is a patented and proprietary modification of a traditional lateral flow device and uses direct sampling microfiltration technology. Two antigen-specific antibodies capture MMP-9 antigens in the sample, and this complex is captured in a proprietary mode at the test result line, giving rise to a visually observable signal [21, 22].

Tear meniscus height (TMH): Tear meniscus height using the Keratograph 5M (Oculus, Germany) topographer will be measured three times consecutively and the median value was recorded [17].

Conjunctival hyperemia (RS score): Conjunctival hyperemia (RS score) will be assessed by Keratograph image (Oculus, Germany) of 1156 × 873 pixels, with redness score (RS) ranges from 0.0 (normal) to 4.0 (severe) [23].

Meibomian quality and meibomian gland expressibility: Meibomian quality will be assessed under a slit-lamp; eight meibomian glands in the middle parts of the eyelid will be assessed using a scale of 0 to 3 for each gland (0 represented clear meibum; 1 represented cloudy meibum; 2 represented cloudy and granular meibum; and 3 represented thick, toothpaste-like consistency meibum). Meibomian gland expression: five meibomian glands in the middle part were evaluated on a scale of 0–3: 0, all glands expressible; 1, 3–4 glands expressible; 2, 1–2 glands expressible; and 3, no glands expressible.

Corneal nerves and immune/inflammatory cell change: HRT III RCM (Heidelberg Engineering GmbH, Germany) will be used to record corneal nerves and immune/inflammatory cell change [24]. A total of 5–8 sequence/volume scans were taken from the center of each cornea, focusing on all corneal layers: superficial, intermediate, and basal epithelial layers; sub-basal nerve plexus, anterior, central, and posterior stroma; and endothelium. Three representative images of the subbasal nerve plexus and epithelial DCs will be selected for analysis for each eye, considering criteria such as a whole image in the same layer, best focus, and good contrast.

OSDI: the Chinese language-validated OSDI, which is a questionnaire consisting of 12 questions for evaluating the effects of dry eye syndrome on vision, ocular symptoms, and any condition associated with DED, will be used [16]. The patient will answer each question on a scale ranging from 0 to 4, with 0 indicating “none of the time” and 4 indicating “all of the time.” If a certain question is deemed irrelevant, it will be marked as “not applicable (N/A)” and excluded from the analysis. The OSDI total score is calculated according to the proprietary formula proposed by the authors of the original OSDI [25]. The scale ranges from 0 to 100, with higher scores representing more severe cases of DED.

### Participant timeline {13}

The schedule for data collection and visits is shown in Fig. 2. After registration for this study, the assigned treatment intervention will be administered for 8 weeks. Furthermore, the effect of eyedrops will be examined during the 4-week and 8-week follow-up period (Fig. 1).

### Sample size {14}

The sample size calculation is based on the primary outcome measures, named NITBUT, to establish the non-inferiority between the groups in terms of the changes in the mean from the baseline at day 56. For the NITBUT, a sample size of 164 subjects, 82 in each group, is sufficient to detect a clinically important difference of 5.24 s [26] between groups in reducing DED assuming a standard deviation of 5.23 s [27] using a two-tailed *t*-test of difference between means with a power of 80% and a level of significance of 5%. Considering a dropout rate of 10% the sample size required is 182 (91 per group). With an additional 10% compensation for the potential deviation of dry eye metrics from a normal distribution [26, 27], the sample size required is 202 (101 per group).

### Recruitment {15}

This study will be conducted between the 1st of March 2023 and the 30th of November 2023. This clinical study will be done in a single site, with participants blinded to the treatment assignment. This research is open to patients diagnosed with DED at He Eye Specialist Hospital’s Department of Ophthalmology. Participants will be recruited using adverts in the distribution pamphlets, the website, and social media postings. To ensure efficient recruitment, the study team members will assist in reaching out to potential participants. Additionally, the coordinator of local community centers for old people will be contacted to aid in recruitment efforts. Each participant’s demographic information (including ocular diseases and current/previous usage of drugs and/or lubricating eye drops) will be collected during the first (screening) appointment. Research participants will consist of subjects diagnosed with DM according to the American Diabetes Association’s 2020 “Standards of Medical Care in Diabetes” [28] and dry eye according to diagnostic criteria set by the Asia Dry Eye Society in 2017 [15]. Participants will not be limited based on age, gender, or ethnicity (Fig. 2). Eligible participants will be notified of the study’s procedures and any further arrangements that need to be made. Recruitment will be stopped when the expected number of patients will be recruited in advance.

### Assignment of interventions: allocation

#### Sequence generation {16a}

Patients will be randomized (using a random number table generated by the Microsoft Excel RANDOM function) in a 1:1 ratio after signing the informed consent to the intervention group or control group.

#### Concealment mechanism {16b}

The block size will be concealed from other researchers and the randomization table will not be available for assessment by anyone else involved in the study [29]. Randomization is performed by an independent biostatistician. The biostatistician is the only one who has access to check the file. The allocation list is kept in a separate file on a separate computer under password protection.

#### Implementation {16c}

Opaque and sealed (a randomization list for each stratum) envelopes containing serial numbers will be prepared by an independent statistician and will be delivered to the clinical trial center.

An independent researcher will distribute the envelopes to participants and allocate them into study groups at a 1:1 ratio, without implementing stratification.

Before the random assignment, all participants will be informed that they will be allocated to one of two groups. Random allocation will be conducted at visit 2. Random numbers with corresponding participants will be determined in the order of the time of the second visit and will be opened by the clinician prescribing the eye drops (DQS or HA). The allocation result will not be announced to the participants until the end of the data collection phase of the trial. Researchers collecting and analyzing data related to this trial will be blinded to the participant allocation results.

### Assignment of interventions: blinding

#### Who will be blinded {17a}

The treatment assignment for the study will be triple-masked. Participants in the research would be unable to recognize the contents. The box containing the ampoules is labeled with a batch number, study reference number, participant ID, contact number, investigator name, site address, the expiration date of the eye drops, storage instructions, and a statement informing the participant that the eye drops are for use only in clinical trials and should not be ingested. Masked examiner for all clinical assessments will not involved in the data collection or group allocation procedure for this research. The investigator will not be aware of the two groups.

#### Procedure for unblinding if needed {17b}

There will be no unblinding procedure in this study.

#### Participant withdrawal

Based on the following criteria, patients will be removed from the research.

1. When it is deemed challenging to continue the study owing to the emergence of new ailments.
2. When the research participant cannot be located.
3. In the case of pregnancy or pregnancy suspicion.
4. When participants want to end their participation in a study.
5. When the participant’s caretaker cannot guarantee their participation in the study.
6. When the research project has concluded.
7. When the lead investigator and sub-investigators believe that it is acceptable to cease the study for reasons other than those listed above.

### Data collection and management

#### Plans for assessment and collection of outcomes {18a}

To prevent data loss, questionnaires will be completed using an online system. Ophthalmic examinations will be performed by experienced ophthalmologists. In order to ensure the accuracy of the results, some tests are averaged three times. Data administration is the responsibility of Jiayan Chen, HESH, Department of Clinical Research, as chosen by the principal investigator (Emmanuel Eric Pazo). This research collects data using a proprietary electronic medical records (EMRs) report form and management application, uploaded to the cloud for backup weekly. Following the database lock, the individual responsible for statistical analysis will get the locked data.

#### Plans to promote participant retention and complete follow-up {18b}

To enhance participant adherence, a research assistant will send regular reminders to use the eyedrop and complete the follow-up. If participants discontinue or deviate from intervention protocols, the study team will initiate contact and prioritize addressing any concerns that may be impacting their adherence to the intervention protocols.

In order to guarantee the completeness and accuracy of the gathered data, the online questionnaires will be encoded in a manner that necessitates respondents to provide comprehensive responses to all inquiries prior to submitting their answers. In addition, to increase motivation to remain in the study, participants will receive transportation subsidies after completion of the follow-up

#### Data management {19}

This study uses the electronic case report form and management tool to collect data. Data collection and data entry were performed by separate experienced staff members at HESH, Department of Clinical Research. Participants will not input their names or any identifying information but will be assigned a unique identifier. To ensure data quality, supervision and double confirmation will be performed by Jiayan Chen, along with weekly backup, ensuring that only authorized users (e.g., the research team members) will have access to the dataset.

#### Confidentiality {27}

Participant’s personal information will be kept confidential in the same way as their medical histories in the hospital before, during, and after the trial. During the course of the research, participants’ personal information will be kept strictly confidential and only accessed by authorized persons only. Participants will be allocated an individual trial identification number and participant details will be stored on a secure password-protected database in the clinical trial center. Following the end of the clinical trial, all participant’s data will be anonymized prior to data analysis and will be available from the corresponding author upon reasonable request.

#### Plans for collection, laboratory evaluation, and storage of biological specimens for genetic or molecular analysis in this trial/future use {33}

There will be no biological specimens collected during the entire trial.

### Statistical methods

#### Statistical methods for primary and secondary outcomes {20a}

In this research, unless otherwise mentioned, the significance level is set to 5% two-sided and the confidence coefficient is set to 95%. The demographic data of the study’s subjects will be tabulated by calculating the mean and standard deviation for continuous variables and the frequency and percentage for categorical variables. If the continuous variables do not follow a normal distribution, they will be converted appropriately by logarithmic transformation or other means and aggregated with the mean and standard deviation, or the median and interquartile range will be utilized as descriptive statistics.

The safety analysis will be conducted based on a safety analysis population that includes subjects who have received at least one dose of medication after randomization.

Data will be collected for all patients participating in the treatment at the following stages: baseline, first follow-up at week 4 (day 28), and second follow-up at week 8 (day 56 ). The primary outcome measures for this study are NITBUT scores before and after treatment. For the main endpoint, between-group comparisons using baseline as a covariate and an analysis of covariance will be done to produce the adjusted mean, its 95% confidence interval, and the *p*-value. A paired *t*-test will be used to make within-group comparisons. To ensure participant safety, frequencies and proportions will be computed for each group and item, and comparisons between groups will be made using Fisher’s exact probability test or the *χ*^2^ statistic.

#### Interim analyses {6}

There will be no planned interim analyses for this study. Since there are no anticipated problems that are detrimental to the participant, so interim analysis is not warranted.

#### Methods for additional analyses (e.g., subgroup analyses) {20b}

Subgroup analyses are not planned for this study.

#### Methods in analysis to handle protocol non-adherence and any statistical methods to handle missing data {20c}

We will use intention-to-treat analysis to analyze the data. Participants who do not follow the protocol are nevertheless considered for the analysis. Missing values will be handled using the best imputation method available.

#### Plans to give access to the full protocol, participant-level data, and statistical code {31c}

The datasets analyzed during the current study and statistical code are available from the corresponding author on reasonable request, as is the full protocol.

### Oversight and monitoring

#### Composition of the coordinating center and trial steering committee {5d}

The subject leader and the project manager will form the Steering Committee (SC). The SC accountable for managing the whole project and having ultimate authority for the study. The SC will write and submit the report for publication at the end of the study.

The Monitor Group’s (MG) inspectors are appointed by the SC. The MG will oversee the entire research procedure in compliance with the GCP requirements. The inspector analyzes the investigator’s adherence to the protocol, the protection of participants’ rights and interests, the quality of the case report form (CRF), and the investigators’ understanding of different standards before submitting inspection reports to the SC.

The principal investigator designated a person (HESH, Department of Clinical Research) to be in charge of the data management. This study uses the Electronic case report form and management tool to collect data.

#### Composition of the data monitoring committee, its role, and reporting structure {21a}

The Department of Clinical Research in HESH to be in charge of the data management The database will be constructed using Excel (Microsoft, USA 2022 version), and regular data monitoring will be undertaken in accordance with the sponsor’s standard operating procedures. The SC manages the trial under the supervision of the MG.

#### Adverse event reporting and harms {22}

Due to the low-risk nature of the trial, no AEs are anticipated. For very rare cases such as eye secretion, eye irritation, eye itching, congestion, and eye pain, HESH Certified Review Board will be notified; experimental treatments will be discontinued promptly, and professional ophthalmologist will provide treatment .

#### Frequency and plans for auditing trial conduct {23}

The trial will be conducted and monitored by the principal investigator. The steering committee will meet weekly to review and evaluate updates.

#### Plans for communicating important protocol amendments to relevant parties (e.g., trial participants, ethical committees) {25}

If there are modifications to eligibility criteria, outcomes, or analyses needed for the study, a revised protocol will be submitted for approval to the Medical Ethics Committee at HESH, Shenyang, China.

#### Dissemination plans {31a}

The study’s findings will be shared regardless of the effect’s direction. All possible beneficiaries of the research, including patients, carers, family, doctors, advisory boards, and medical boards, will receive trial data. Publications in high-impact, open-access medical journals and talks at national and international medical conferences will serve this purpose.

## Discussion

DM is a complex metabolic disorder and several mechanisms have been speculated for the cause of DE in DM patients. Some suggest that insulin is necessary for the proliferation of acinar lacrimal gland and cornea epithelial cells [30], while others propose that the tear reflex is weakened owing to diabetic corneal neuropathy-induced corneal insensitivity [31]. Additionally, it has been shown that the amount of glucose and advanced glycation end-products (AGEs) might rise in tears, promoting hyperosmolarity and inflammation [32].

Prior studies have shown that DM increases the risk of developing chronic DED [33, 34] due to tear protein alteration, decreased corneal sensitivity, and lower reflex-induced tear secretion [35]. Manaviat et al. [36] observed that the prevalence of DED in type 2 diabetic patients was 54.3% and that the rate of morbidity was significantly greater than in non-diabetic controls. In addition, they discovered that DED was substantially linked with age, gender, diabetes duration, and diabetic retinopathy. Studies conducted by Najafi et al. [37] and Zou et al. [34] suggest that glycosylated hemoglobin (HbA1c) levels had an association with diabetic DED.

DQS stimulates P2Y2 receptors on the ocular surface, which enhances the secretion of water and secretory mucin from conjunctival tissue. Therefore, tear film stability and quality can be improved [38]. However, the effect of DQS on the tear film of DM humans has not been previously assessed. This result should allow us to compare the clinical efficacy and safety of 3% DQS in diabetic patients with DED and provide clinicians with more treatment options. On the other hand, HA is a glycosaminoglycan, and with its sponge-like structure of polysaccharide chains, it can bind with water and acts as a reservoir for slowly releasing water molecules [39]. As a result, HA eye drops are commonly used to treat DED, while initial findings from a pilot study suggest that HA eye drops can improve signs and symptoms of diabetic DE [40]. The possible benefits exhibited by the two topical ophthalmic eyedrops will be related to corneal epithelial recovery and secretion and not changes in the underlying pathology created due to DM.

However, a large RCT has not been conducted to understand the benefits of HA eye drops on diabetic DED patients.

## Trial status

Recruitment began in May 2023 and the approximate date when recruitment will be completed is August 2023. Protocol version 3.0 was approved in Dec. 2022.

### Supplementary Information

**Additional file 1.**

## Abbreviations

AGEs: Advanced glycation end-products
AEs: Adverse events
ATDs: Artificial tear drops
BCVA: Best-corrected visual acuity
CS: Conjunctivocorneal staining
CRF: Case report form
DED: Dry eye disease
DM: Diabetes mellites
DK: Diabetic keratopathy
DQS: Diquafosol
EMRs: Electronic medical records
GCP: Good clinical practice
HESH: He Eye Specialist Hospital
HA: Hyaluronic acid
HRT III: Heidelberg retina tomography III
IOP: Intraocular pressure
MG: Monitor Group
NITBUT: Noninvasive tear breakup time
OSDI: Ocular Surface Disease Index
PI: Principal investigator
RCT: Randomized control trial
RS: Conjunctival hyperemia
SOPs: Standard operating procedures
SC: Steering Committee
TFLL: Tear film lipid layer
THM: Tear meniscus height
