# Supplementary material for: A protocol for a single center, randomized, controlled trial comparing the clinical efficacy of 3% diquafosol and 0.1% hyaluronic acid in diabetic patients with dry eye disease
Source: Trials. 2023 Dec 12;24:803. doi: 10.1186/s13063-023-07818-8 (PMC10714480; doi:10.1186/s13063-023-07818-8)
Supplement: Supplementary file 1 — Additional file 1. [file 13063_2023_7818_MOESM1_ESM.docx]

***Trials* structured Study Protocol template** *Trials* guidance: the numbers in curly brackets (e.g. {5a}) are SPIRIT item identifiers. **Please do not remove the numbers in curly brackets, or any heading that contains them**. The item identifiers are slightly out of sequence to make the document flow more easily, but it is important that they remain in the document to allow electronic searches by SPIRIT item number.

If you are certain that an item does not apply, please state "n/a" and provide a short explanation. Leaving an item blank or stating “n/a” without an explanation will lead to your manuscript being returned before review.

NB: All text in green can be removed once you have finished creating your Study Protocol. Text in black is mandatory. Please also read the submission guidelines for Study Protocols prior to submitting to *Trials*: [https://trialsjournal.biomedcentral.com/submission-guidelines/preparing-your-manuscript/study-protocoll](https://trialsjournal.biomedcentral.com/submission-guidelines/preparing-your-manuscript/study-protocol)

**CHECKLIST**

**Title:** *Line 3*

**Names protocol contributors:** *Line 6*

**Abstract:** *Line 18*

- **Background**: *Line 19*
- **Methods**: *Line 29*
- **Discussion**: *Line 37*

**Trial registration**: *Line 41*

**Keywords:** *Line 45*

**Administrative information:** *Line 55*

Note: the numbers in curly brackets in this protocol refer to SPIRIT checklist item numbers. The order of the items has been modified to group similar items (see <http://www.equator-network.org/reporting-guidelines/spirit-2013-statement-defining-standard-protocol-items-for-clinical-trials/>).

| Title {1}: *line 55* | SPIRIT guidance: Descriptive title identifying the study design, population, interventions, and, if applicable, trial acronym. |
| --- | --- |
| Trial registration {2a and 2b}. *line 55* | SPIRIT guidance: Trial identifier and registry name. If not yet registered, name of intended registry.  Item 2b is met if the register used for registration collects all items from the World Health Organization Trial Registration Data Set. |
| Protocol version {3} *line 55* | SPIRIT guidance: Date and version identifier. |
| Funding {4} *line 55* | SPIRIT guidance: Sources and types of financial, material, and other support. |
| Author details {5a} *line 55* | SPIRIT guidance: Affiliations of protocol contributors. |
| Name and contact information for the trial sponsor {5b} *line 55* | SPIRIT guidance: Name and contact information for the trial sponsor. |
| Role of sponsor {5c} *line 55* | SPIRIT guidance: Role of study sponsor and funders, if any, in study design; collection, management, analysis, and interpretation of data; writing of the report; and the decision to submit the report for publication, including whether they will have ultimate authority over any of these activities. |

**Introduction**

**Background and rationale {6a}:** *Line 60*

**Objectives {7}:** *Line 103*

**Trial design {8}:** *Line 108*

**Methods: Participants, interventions and outcomes**

**Study setting {9}:** *Line 118*

**Eligibility criteria {10}:** *Line 124*

**Who will take informed consent? {26a}:** *Line 164*

**Additional consent provisions for collection and use of participant data and biological specimens {26b}:** *Line 168*

**Interventions**

**Explanation for the choice of comparators {6b}:** *Line 174*

**Intervention description {11a}**: *Line 185*

**Criteria for discontinuing or modifying allocated interventions {11b}:** *Line 192*

**Strategies to improve adherence to interventions {11c}:** *Line 202*

**Relevant concomitant care permitted or prohibited during the trial {11d}:** *Line 210*

**Provisions for post-trial care {30}:** *Line 214*

**Outcomes {12}:** *Line 220*

**Participant timeline {13}**: *Line 283*

**Sample size {14}:** *Line 290*

**Recruitment {15}:** *Line 299*

**Assignment of interventions: allocation**

**Sequence generation {16a}:** *Line 314*

**Concealment mechanism {16b}:** *Line 319*

**Implementation {16c}:** *Line 326*

**Assignment of interventions: Blinding**

**Who will be blinded {17a}:** *Line 341*

**Procedure for unblinding if needed {17b}:** *Line 349*

**Data collection and management**

**Plans for assessment and collection of outcomes {18a}:** *Line 366*

**Plans to promote participant retention and complete follow-up {18b}:** *Line 376*

**Data management {19}:** *Line 391*

**Confidentiality {27}:** *Line 397*

**Plans for collection, laboratory evaluation and storage of biological specimens for genetic or molecular analysis in this trial/future use {33}:** *Line 409*

**Statistical methods**

**Statistical methods for primary and secondary outcomes {20a}:** *Line 413*

**Interim analyses {21b}:** *Line 429*

**Methods for additional analyses (e.g. subgroup analyses) {20b}:** *Line 335*

**Methods in analysis to handle protocol non-adherence and any statistical methods to handle missing data {20c}:** *Line 437*

**Plans to give access to the full protocol, participant level-data and statistical code {31c}:** *Line 445*

**Oversight and monitoring**

**Composition of the coordinating centre and trial steering committee {5d}:** *Line 452*

**Composition of the data monitoring committee, its role and reporting structure {21a}:** *Line 463*

**Adverse event reporting and harms {22}:** *Line 480*

**Frequency and plans for auditing trial conduct {23}:** *Line 488*

**Plans for communicating important protocol amendments to relevant parties (e.g. trial participants, ethical committees) {25}:** *Line 492*

**Dissemination plans {31a}:** *Line 498*

**Discussion:** *Line 506*

**Trial status:** *Line 538*

**Abbreviations:** *Line 542*

**Declarations:** *Line 554*

- Acknowledgements
- Authors' contributions
- Funding
- Availability of data and material
- Ethics approval and consent to participate
- Consent for publication
- Competing interests
- Authors’ information (optional)

**Acknowledgements:** *Line 556*

**Authors’ contributions {31b}:** *Line 560*

**Funding {4}:** *Line 567*

**Availability of data and materials {29}:** *Line 572*

**Ethics approval and consent to participate {24}:** *Line 575*

**Consent for publication {32}:** *Line 583*

**Competing interests {28}:** *Line 590*

**Authors’ information (optional):** *Line 592*

**References:** *Line 605*
